# The impact of mycobacteria-induced trained immunity on SARS-CoV-2 vaccine responses

**DOI:** 10.3389/fimmu.2025.1633977

**Published:** 2025-09-03

**Authors:** Lidia Sánchez-Morales, Néstor Porras, Andrea Pérez-Domingo, Marta Pérez-Sancho, Teresa García-Seco, Marta Diaz-Frutos, Aranzazu Buendia, Inmaculada Moreno, Leydis Zamora, Ana Balseiro, M. A. Risalde, Antonio Rodriguez-Bertos, Christian Gortázar, Mercedes Domínguez, Lucas Domínguez

**Affiliations:** ^1^ VISAVET Health Surveillance Centre, Complutense University of Madrid, Madrid, Spain; ^2^ Department of Animal Health, Faculty of Veterinary Medicine, Complutense University of Madrid, Madrid, Spain; ^3^ Immunology Unit, National Microbiology Centre, Carlos III Health Institute, Majadahonda, Madrid, Spain; ^4^ Department of Animal Health, Faculty of Veterinary Medicine, University of León, León, Spain; ^5^ Instituto de Ganadería de Montaña (IGM, CSIC-ULE), León, Spain; ^6^ Department of Comparative Anatomy and Pathology and Toxicology, Animal Health and Zoonosis Research Group (GISAZ), UIC Zoonosis and Emerging Diseases (ENZOEM), University of Cordoba, Córdoba, Spain; ^7^ Department of Internal Medicine and Animal Surgery, Faculty of Veterinary Medicine, Complutense University of Madrid, Madrid, Spain; ^8^ SaBio Instituto de Investigación en Recursos Cinegéticos IREC (CSIC-UCLM-JCCM), Ciudad Real, Spain

**Keywords:** trained immunity, SARS-CoV-2 vaccine, mycobacteria, innate memory, adaptative immunity

## Abstract

**Introduction:**

Beyond the role of Bacillus Calmette-Guérin (BCG) for tuberculosis prevention, BCG has demonstrated heterologous protective effects. The global health crisis caused by the SARS-CoV-2 virus led to research on whether BCG-induced trained immunity could strengthen antiviral defenses. However, studies reported quite different results on its effect against COVID-19.

**Methods and results:**

In this study, we evaluated the impact of pre-existing trained immunity induced by a BCG-derived *Mycobacterium bovis* strain (dpB), in both live and inactivated forms, in combination with SARS-CoV-2 vaccination prior to challenge in a mouse model. While the SARS-CoV-2 vaccine was enough for protection in morbidity and mortality terms, its combination with live dpB significantly enhanced immune responses reflected in higher levels of pro-inflammatory cytokines, reduced pulmonary viral loads, and improved histopathological outcomes. Additionally, the formation of inducible bronchus-associated lymphoid tissue (iBALT) in lungs in vaccinated animals pre-exposed to live dpB points to a potential mechanism for long-term immune surveillance in the respiratory tract.

**Conclusions:**

These immunological findings highlight the potential benefits of integrating trained immunity inducers with pathogen-specific vaccines to enhance immune responses and protection. Further research is needed to optimize immunomodulation strategies, dosing regimens and administration routes to maximize these synergistic effects and prevent potential negative effects.

## Introduction

Trained immunity (the ‘memory’ of innate immune response) is associated with the induction of a series of epigenetic and metabolic changes in the cells of the innate immunity that lead to the activation and differentiation of macrophages, the production of neutrophils, and increased T and B lymphocyte activity (1, 2). Epigenetic changes result in greater transcription of genes and higher production of cytokines such as TNF-α, IL-6, and IL-1β up to three months after the first BCG dose (3, 4). All these epigenetic and metabolic changes involve the ability of the innate immune system to remember and respond in an enhanced manner to previous stimuli (2).

Innate memory and adaptive response represent two pillars of the immune system that could interact synergistically to provide a more robust and efficient defense against different pathogens. Trained immunity may complement adaptive immunity, which relies on the specificity and long-term memory of T and B lymphocytes (4). In this way, ‘traditional vaccines’, designed primarily to stimulate adaptive immunity, can benefit from the mechanisms of trained immunity to enhance their efficacy. For example, vaccines incorporating adjuvants capable of inducing trained immunity could offer broader and longer-lasting protection. However, these not only induce immune response against the specific pathogen for which they are designed, but also against heterologous infections (5, 6). On this basis, some experimental studies have examined the combination of products known as trained immunity-based vaccines (TIbV) such as MV140 (7) or MV130 (8). They both are heat inactivated bacteria mixtures, with vaccines targeting genitourinary tract infections (7) or SARS-CoV-2 (8), potentially leading to improved outcomes.

Beyond the specific role of Bacillus Calmette Guerin (BCG) vaccine in tuberculosis control, it has been associated with non-specific protective effects against various infections, including viral pathogens. Several clinical studies reported non-specific effects of BCG against heterologous respiratory infections both in children (5, 6) and in the elderly (9). Also, experimental studies reported protection against herpes virus (10), influenza virus (11), Leishmania major (12), Plasmodium spp. (13) or Candida albicans (14) in murine models. Regarding SARS-CoV-2, there are contrasting experimental and epidemiological studies concerning the impact of prior BCG stimulation on COVID-19, with observations ranging from protection (15–19) to lack of protection (20–23) or even negative effects (24, 25). Besides, studies in which BCG has been used as an adjuvant to the COVID19 vaccine have also been carried out, demonstrating higher immunogenicity (26).

Given the widespread use of BCG for tuberculosis vaccination, its potential influence on the immune response to emerging viral threats such as SARS-CoV-2 is of particular interest. Unlike previous studies which used immunomodulators as adjuvants (26), our hypothesis is that previous contact with some mycobacteria (in our case, dpB, a Danish CCUG 27863 BCG-strain derived from porcine) may modulate the acquired immune response induced by vaccines. In this case, those against SARS-CoV-2 to improve the pathogen-specific protection (27). The use of dpB was also motivated by our group’s prior work on this strain which has proved immunomodulatory efficacy in various models (*data not shown*). The dpB derivative, which shows a high homology with Danish CCUG 27863, obtained from swine and subsequent formulation, has showed relevant immunostimulatory properties. In addition, we aimed to assess the importance of the format of the dpB considering how different BCG formulations can modify the result as previously shown regarding the sequential use of live BCG and inactivated *M. bovis* (28). We investigate the response, effectiveness and protection of the combination of prior dpB inoculation to specific vaccination against SARS-CoV-2 to assess the possible synergistic effect between the training of innate immunity and the induction of acquired immunity, evaluating also the impact of the dpB format (live and inactivated) on its immunomodulator effect.

## Materials and methods

### Animals

Animal care and procedures were performed by following the guidelines of good experimental practices according to Directive 2010/63/EU of the European Parliament and of the Council of 22 September 2010 on the protection of animals used for scientific purposes [amended by the Regulation (EU) 2019/1010)] and Spanish laws (RD 53/2013). The protocol was approved by the Community of Madrid Ethics Committee (reference PROEX 180.2/22). K18-hACE2 mice strain, expressing angiotensin-converting enzyme 2 (hACE2), necessary for SARS-CoV-2 to enter the host cells, was used in order to evaluate the effectiveness of dpB in combination with specific SARS-CoV-2 vaccine against SARS-CoV-2 infection. K18-hAC2 female mice (n=108) aged 4–6 weeks were obtained from Charles River Laboratories España S.A (Sant Cugat del Vallès, Barcelona, Spain). The cages were equipped with Altromin-LASQCdiet^®^ Rod 14-H (Altromin Lage, Germany) for feeding. Both food and water were available *ad libitum*. Wheeled houses for environmental enrichment were also included (Ref: K3327 + K3250, Sodispan Research, Madrid, Spain). During the initial 2-week period, they were given time to acclimate to their new cages and socialize with their partners. After that period, the animals were marked with an ear tagger for individual identification (in many cases, it was not necessary since mice were easily identifiable by white areas on their tails). DpB and vaccine administration procedures were carried out in the Biosafety Level 2 + (BSL2+) area at the VISAVET Health Surveillance Centre (Complutense University, Madrid). Afterwards, animals were moved to the Biosafety Level 3 (BSL3) area at the VISAVET Centre for SARS-CoV-2 infection studies.

### SARS-CoV-2 virus and cell lines

SARS-CoV-2 MAD6 was used for experimental infection assay. Calu-3 cells were prepared to reproduce stocks of SARS-CoV-2 (29). The cells were incubated at 37 °C under 5% CO_2_ in Eagle’s Minimum Essential Medium (EMEM) with L-glutamine (Merck KGaA, Darmstadt, Germany) and supplemented with 100 IU/mL penicillin, 100 μg/mL streptomycin, and 10% fetal bovine serum (FBS) (Merck KGaA, Darmstadt, Germany).

For viral growth in Calu-3 cells, a multiplicity of infection (MOI) of 0.0001 was used. After the virus was absorbed for one hour at 37°C, the viral growth media EMEM supplemented with 2% fetal bovine serum was added. The cell lysate and supernatant were harvested after 3 days of incubation at 37 °C with 5% CO_2_.

Vero E6 cells, provided by the Carlos III Health Institute (Madrid, Spain), or ATCC^®^ (Manassas, Virginia, USA), were prepared to titrate SARS-CoV-2 stocks by determining the amount of virus causing cytopathic effects in 50% of tissue culture infectious dose (TCID50/mL). Additionally, this cell line was used to verify the viability of the SARS-CoV-2 inoculum used for infecting the animals after the procedure.

### DpB preparation

A frozen vial of dpB was inoculated in a starter liquid culture medium and incubated at 37 °C in aerobiosis for 4 weeks and then inoculated in a liquid culture medium at 37 °C in aerobiosis for another 4 weeks. At the end of the culture, the growth was collected with a pipette, centrifuged, washed with PBS and given a treatment of physical breakage with glass beads, and diluted with PBS until a homogeneous product was at approximately 1 McFarland [equivalent to approximately 10^6^ colony forming units (CFU)/mL]. The final dose for inoculation obtained was 6.3x10^5^ CFU/mL.

For dpB inactivation, formaldehyde (37%) is added until achieving a final concentration of 0.1%. The growth is immersed in a bath of 75 °C for 70 minutes and afterwards placed on a sonicator (Misonix Sonicator XL2020 Ultrasonic Liquid Processor), conducting during 10 cycles that included 3 minutes of sonication and 1 minute of pause. The dpB concentration of the final solution was then adjusted to 1.2 McF (10^6^ CFU/mL) with PBS.

### SARS-CoV-2 vaccine preparation

Vaccine employed in the first administration was combined with a complete Freund adjuvant (inactivated mycobacterium). Vaccine employed in the second administration (boost) was combined with incomplete Freund adjuvant (30).

### Experimental design

**Figure 1:**
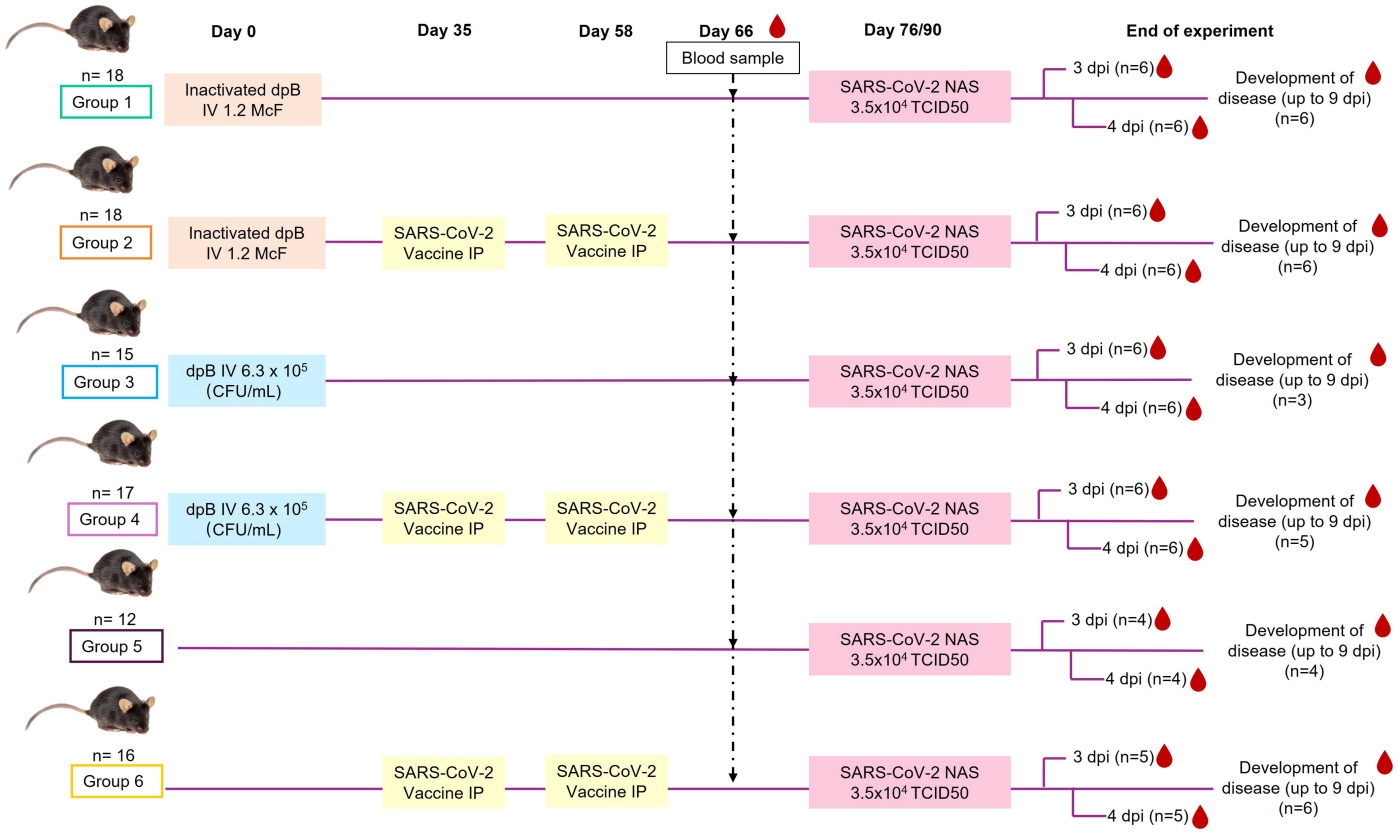
Experimental design and timeline, depicting the stimulation-to-sacrifice period for the different groups of animals included in the study (IV, intravenous; NAS, intranasal; IP, intraperitoneal; dpi, day post infection; CFU, colony forming units.

Animals were divided into 6 different groups (**Figure 1**) each of them with a different treatment: 1) Inactivated dpB, 2) Inactivated dpB - SARS-CoV-2 vaccine, 3) dpB, 4) dpB - SARS-CoV-2 vaccine, 5) Infection control, and 6) SARS-CoV-2 vaccine. Inoculation of inactivated dpB and live dpB took place on day 0, followed by SARS-CoV-2 vaccine on day 35 and its boost on day 58 of the experiment. All the animals were then challenged with SARS-CoV-2 on day 76 or 90. All the groups were divided into two subgroups following-up duration: i) animals sacrificed 3–4 days post infection (dpi) and ii) animals that developed the disease. Animals were sacrificed using cervical dislocation when reaching the endpoint criteria described in the following section (5, 6, 7, 8 dpi), or animals left until the 9 dpi (end of the experiment) that did not develop COVID-19 or did not reach endpoint criteria.

### DpB administration, SARS-CoV-2 infection, and sampling

DpB both alive (6.3 x 105 CFU/mL) and inactivated (1.2 McF) were intravenously (IV) administered (100 µl per mice) 2 weeks after the arrival of the mice, using a 30 G needle through the caudal vein of the tail (groups 1, 2, 3 and 4). A restrainer was used to immobilize the mice and the tail was pre-warmed slightly using a heat lamp for enhanced vasodilation for dpB administration.

SARS-CoV-2 vaccine was administered intraperitoneally (IP) (200 µL) in two different times. First inoculation was carried out 35 days after dpB-stimulation and the vaccine boost was administered 22 days after the first inoculation.

A blood sampling in 8–9 animals from each group was carried out 2 months after dpB stimulation and before SARS-CoV-2 infection. A total blood volume of 200-250 µL was obtained throughout venipuncture with a 23 G needle. Gauze was used for compression to stop the bleeding and once the bleeding stopped, IP physiological serum was administered to replace fluids. Subsequently, plasma was obtained for the detection of antibodies against SARS-CoV-2, inflammation and coagulation markers and the determination of cytokines (**Figure 1**).

Animals were challenged with SARS-CoV-2 virus 76 to 90 days after the dpB administration (**Figure 1**). For infection, the 101 mice (groups 1-6) were sedated with xylazine (20 mg/mL) [Xilagesic 20MG 25ML, CALIER, Les Franqueses del Vallès, Barcelona] at a dose of 2 mg/kg and ketamine 100 mg/mL [Ketamidor 100 mg/ml, 50 ml, richter pharma, Austria] at a dose of 20 mg/kg intraperitoneally (IP). Afterwards, animals were inoculated intranasally (NAS) with SARS-CoV-2 at a dosage of 5x104 PFUs or 3.5x104 TCID50 per mouse. The total volume inoculated was 25 μL alternating nostrils in volumetric fractions of 5 μL (**Figure 1**).

To confirm that animals were successfully infected, oropharyngeal swab samples were collected at 2 dpi [DeltaSwab^®^ Virus with viral transport media (MTV) (Deltalab S.L., Cataluña, Spain)].

Following the challenge, animals were weighed and monitored daily for clinical signs. A clinical scoring table (**Table 1**) was prepared and utilized to document each clinical sign on a scale from 0 to 2 by two different operators, so the subjectivity was lowed to the minimum. The animals were sacrificed upon reaching a cumulative clinical score (sum of scores for each evaluated clinical sign) of 5 or a loss of weight higher than 20%. Once the designated sacrifice days were reached (3 and 4 dpi) or endpoint criteria were fulfilled, blood samples in heparin were obtained via cardiopuncture from the animals after sedation IP with xylazine (20 mg/mL) at a dose of 4 mg/kg and ketamine (100 mg/mL) at a dose of 40 mg/kg, before sacrifice. The animals that did not meet the endpoint criteria were euthanized on 9 dpi, marking the end of the experiment. Subsequently, comprehensive necropsy was conducted on the animals. In this study, brain, upper respiratory tract (trachea and nasal turbinates), lower respiratory tract (lungs) and heart samples were collected in AllProtect Tissue Reagent (Qiagen, Venlo, Netherlands) for viral RNA detection and quantification. The former tissues were also collected in 10% neutral formalin for further histopathological studies.

**Table 1:** Score of the different clinical signs analyzed throughout the experience daily since the infection of the animals.

Clinical score	0	1	2
*Loss of weight*	None/Slight (0-10%)	Moderate (10-20%)	Severe (>20%)
*Hair appearance*	Undamaged	Slightly tousled	Bristly
*Level of activity*	Normal	Reduced activity	Inactive
*Eye closing*	Normal eyes	Slightly bent	Totally closed
*Respiratory signs*	Normal	Slightly increased	Tachypnoea, dyspnea or abdominal tightening
*Neurological signs*	None	Depression, bending posture, difficulty in walking	Tremors or convulsions

### SARS-CoV-2 RNA extraction and reverse transcription-quantitative PCR (R

MagMAX™ CORE Nucleic Acid Purification Kit was used for RNA extraction of tissues according to the manufacturer’s instructions with the KingFisher Flex (Thermofisher) automatized system. In addition, the detection and quantification of SARS-CoV-2 loads from tissues and oropharyngeal swabs was performed using the CoVID19 dtec RT qPCR Test (Genetic PCR Solutions™, Alicante, Spain) including the respective positive and negative controls.

### SARS-CoV-2 neutralizing antibodies

SARS-CoV-2 neutralizing antibodies were detected in all samples using the GenScript cPass™ SARS-CoV-2 Neutralizing Antibody Detection Kit (GenScript Inc., Piscataway, NJ, United States). In this procedure, the protein-protein interaction between HRP-RBD (horseradish peroxidase-receptor binding domain) and human angiotensin-converting enzyme II (hACE2) can be blocked by neutralizing antibodies against SARS-CoV-2 RBD, which was previously validated (31). Samples presenting a cutoff higher or equal to 30% were considered positive, indicating the presence of SARS-CoV-2 neutralizing antibodies, and samples with a cut-off lower than 30% were considered negative according to the manufacturer’s instructions.

### Measurements of inflammatory and coagulation markers

Complete heparinized blood was centrifugated and plasma was stored at -80 °C until the analyses of the following biomarkers: CRP (C-reactive protein) (Mouse CRP ELISA Kit. Invitrogen, Massachusetts, USA), D-dimer [Mouse D2D (D-Dimer) ELISA Kit. FineTest^®^, Boulder, USA] and iNOS (nitric oxide synthase) (iNOS ELISA Kit. MyBioSource, San Diego, USA).

### Cytokine measurements

Proinflammatory cytokines as interleukin 1β (IL-1β), interleukin 6 (IL-6), tumor necrosis factor alpha (TNF-α), interferon gamma (IFN-γ), and anti-inflammatory as interleukin 10 (IL-10) and transforming growth factor beta (TGF-β) were measured in plasma with the ProcartaPlex™ System (Invitrogen, Waltham, MA, USA) and Bio-Plex 200 equipment (Bio-Rad Laboratories, Alcobendas, Madrid, Spain). These measurements were made both before SARS-CoV-2 infection in 8–9 animals per group 2 months after BCG stimulation and in all the animals post-SARS-CoV-2 infection in the moment of the sacrifice of each animal.

### Histopathological evaluation

Tissues were fixed in 10% neutral formalin (Panreac AppliChem ITW Reagents, Barcelona, Spain) for 48 hours. The samples were automatically processed (Citadel 2000 Tissue Processor, Thermo Fisher Scientific, Waltham, MA, USA) and embedded in paraffin (HistoStar Embedding Workstation, Thermo Fisher Scientific, Waltham, MA, USA). Serial sections of 4 µm thickness were obtained using a microtome (FinesseMe+, Thermo Fisher Scientific, Waltham, MA, USA), and stained with hematoxylin-eosin (HE) (Gemini AS Automated Slide Stainer, Thermo Fisher Scientific, Waltham, MA). Finally, samples were mounted (CTM6 Coverslipper, Thermo Fisher Scientific) and evaluated for histopathological alterations under a Leica DM2000 microscope (Leica Microsystems, Wetzlar, 162 Germany). A single-blind study was conducted by the pathologist in which different lesions of brain and lungs (indicated in **Supplementary Table S1**) were evaluated using a histological score, which assigned a value between 0–3 based on severity.

### Statistical analysis and graphic creation

Initially, normality tests (Shapiro-Wilk) were conducted, revealing that the distribution was not normal for most variables. Differences between groups for each of the variables were evaluated using Kruskal-Wallis and Mann-Whitney test in SPSS (version 28.0.1.1). Mann-Whitney p-values were adjusted by Borferroni method (p_b_). The results were considered statistically significant when the p_b_-value was less than 0.05. Furthermore, a correlation analysis of the different variables was carried out using Spearman’s test.

**Figure 2:**
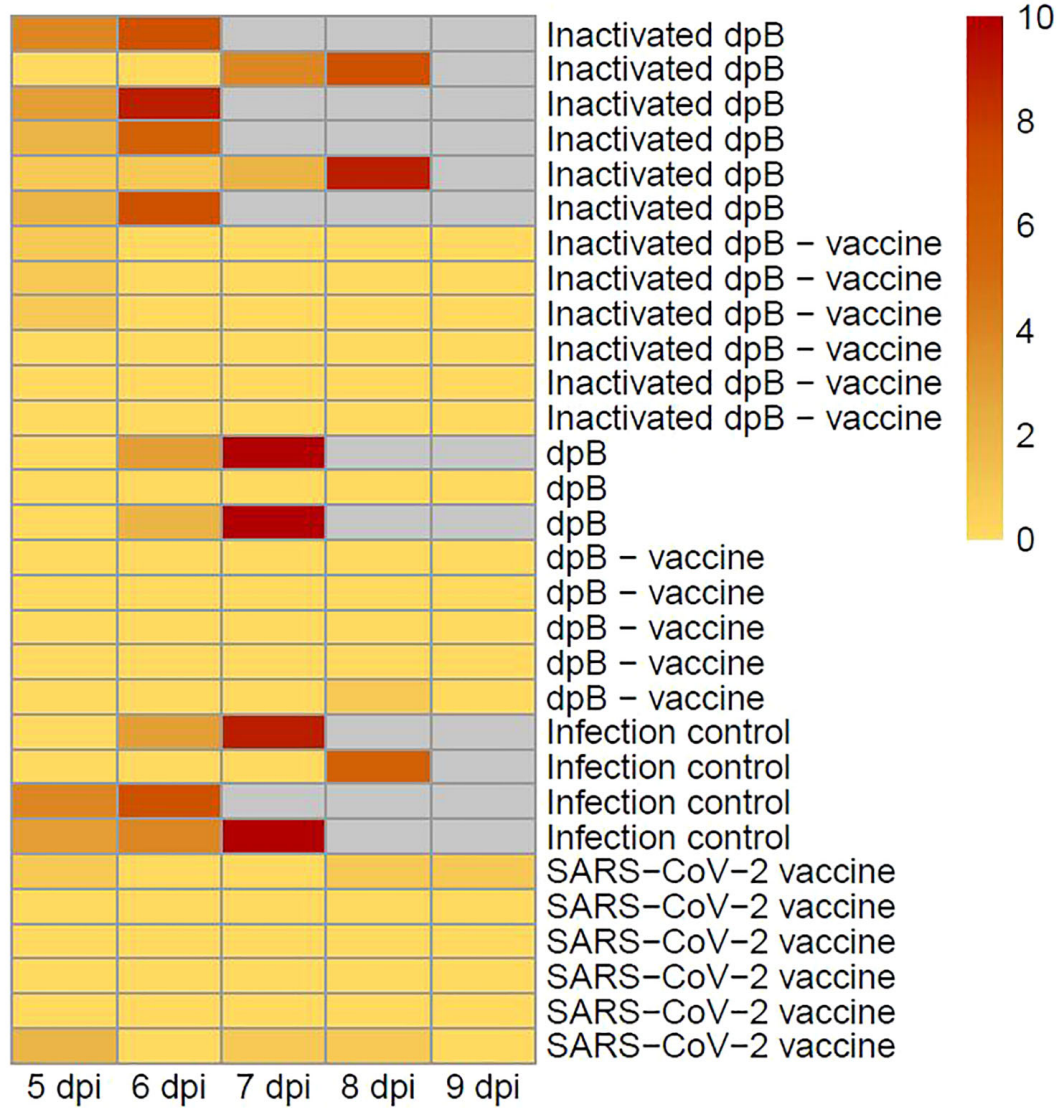
Clinical score associated with clinical signs represented by colors for all the animals in the different groups after SARS-CoV-2 infection: Inactivated dpB (group 1), inactivated dpB-SARS-CoV-2 vaccine (group 2), dpB (group 3), dpB-SARS-CoV-2 vaccine (group 4), infection control (group 5), SARS-CoV-2 vaccine (group 6). They are represented from the 5^th^ day post infection (dpi) (the day on which clinical signs began to be seen in some animals) to 9 dpi (date of final sacrifice of the experiment). The grey boxes represent that the animal had already been slaughtered, so no clinical score is shown for that day or the following days.

All graphical representations included in this study were created using R (version [4.4.2]) within the RStudio integrated development environment (IDE) (32). The ggplot2 package (33) was used to generate scatter plots, boxplots, and jittered point overlays, while the heatmap package (34) was employed for heatmap visualizations (**Figure 2**). Custom color palettes and additional annotations were applied to ensure clarity and high-quality presentation of the data.

## Results

### SARS-CoV-2 vaccine protected animals in terms of clinical signs by itself

Mortality was 0% in all groups vaccinated against SARS-CoV-2 (groups 2, 4, 6), regardless of prior dpB stimulation, with only minimal clinical signs observed, such as slight weight loss or tousled hair (**Figure 2**). This underscores the efficacy of the SARS-CoV-2 vaccine employed in this study in preventing severe disease, even without additional immunomodulation. Thus, those animals that had been vaccinated were positive for neutralizing antibodies in the first sampling before infection (31 days after first dose of SARS-CoV-2 vaccine), as well as in post-infection sampling. However, the animals with no SARS-CoV-2 vaccine, started to develop antibodies after 6–7 dpi (data not shown).

### Higher pre-infection IFN-γ response was induced in dpB - SARS-CoV-2 vaccinated animals

**Figure 3:**
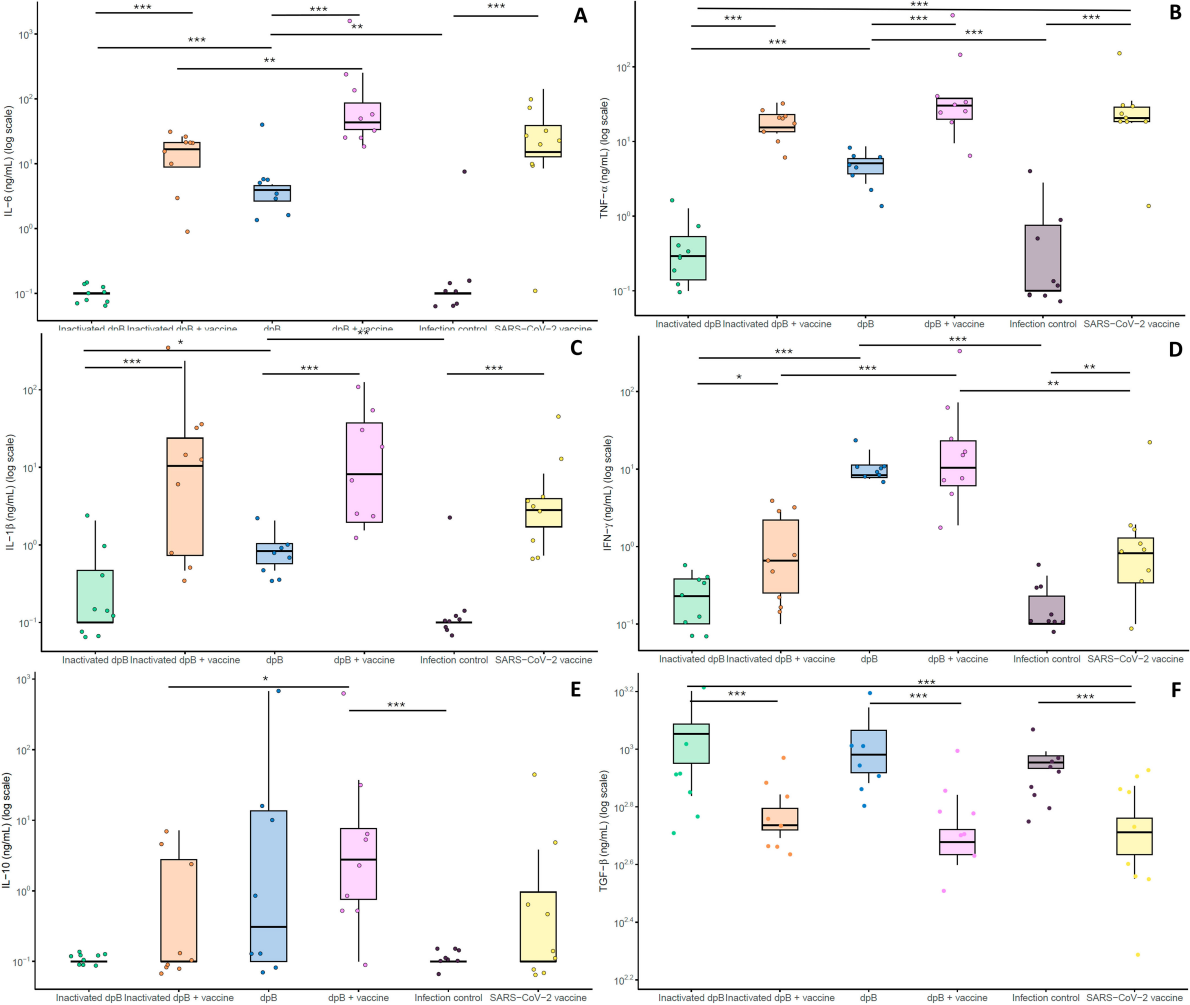
Graphical logarithmical representation (log ng/ml) for pre-infection cytokines (day 66 after first dose of dpB) both pro-inflammatory: IL-6 **(A)**, TNF- α **(B)**, Il-1β **(C)**, IFN-γ **(D)** and anti-inflammatory cytokines: IL-10 **(E)**, TGF-β **(F)** for each of the experimental groups: Inactivated dpB (group 1), inactivated dpB - SARS-CoV-2 (group 2), dpB (group 3), dpB - SARS-CoV-2 vaccine (group 4), infection control (group 5) and SARS-CoV-2 vaccine group (group 6). Statistical significance is indicated as follows: ***p ≤ 0.001, **p ≤ 0.01, *p < 0.05.

In pre-infection samples (day 66), animals receiving the combination of live dpB and SARS-CoV-2 vaccination (group 4) showed statistically higher levels of IL-6 and IL-10, than inactivated dpB-SARS-CoV-2 vaccine group (group 2) (M-W, p_b_< 0.001; M-W, pb=0.040) (**Figures 3A, E**). Also, group 4 exhibited higher levels of IFN-γ cytokine compared to group 2 (M-W, p_b_<0.001) and to SARS-CoV-2 vaccinated-only animals (group 6) (M-W, p_b_=0.003) (**Figure 3D**). Similarly, for almost all cytokine levels (IL-6, TNF-α, IL-1β and TGF-β) significant differences with higher values were observed between animals with SARS-CoV-2 vaccine (groups 2, 4, 6) and the respective non-vaccinated groups (groups 1, 3 and 5) (M-W, p<0.05). Significant differences were observed for animals with inactivated dpB and SARS-CoV-2 vaccine versus inactivated dpB, live dpB and SARS-CoV-2 vaccinated group versus only live dpB group and SARS-CoV-2 vaccine group versus the infection control group (M-W, p<0.05).

For assessing the cytokine response, negative correlations were observed (Spearman test) between pre-infection levels of pro-inflammatory cytokines (IL-1β, TNF-α, IL-6) and viral loads in the moment of sacrifice in multiple organs, including the brain, lungs, and heart (**Table 2**). Interestingly, a similar negative correlation was found for anti-inflammatory cytokine IL-10 and viral loads in the lungs, and for IFN-γ in both lungs and heart, reinforcing the protective role of these cytokines in viral clearance (**Table 2**). Conversely, pre-infection TGF-β levels positively correlated with higher viral loads in the brain, lungs, and heart, potentially suggesting a suppressive effect on immune activation that limits effective viral control (**Table 2**).

**Table 2:** Numerical correlations observed for pre-infection cytokines measured in plasma (IL-1β, TNF-α, IL-6, IL-10, IFN-γ and TGF-β) and viral loads in brain, lungs and heart for animals from all the groups of the experiment.

Tissues	IL-1β	TNF-α	IL-6	IL-10	IFN-γ	TGF-β
Brain	p=0.002** ρ= -0.421	p<0.001*** ρ= -0.443	p=0.002** ρ= -0.421	p=0.211 ρ= -0.176	p=0.057 ρ= -0.263	p=0.042* ρ= 0.295
Lungs	p<0.001*** ρ= -0.590	p<0.001*** ρ= -0.742	p<0.001*** ρ= -0.729	p=0.007** ρ= -0.367	p<0.001*** ρ= -0.480	p<0.001*** ρ= 0.607
Heart	p=0.013* ρ= -0.343	p=0.001*** ρ= -0.431	p=0.002** ρ= -0.419	p=0.103 ρ= -0.229	p=0.026* ρ= -0.305	p=0.019* ρ= 0.338

p values as well as Spearman`s Rho (ρ) are shown. Statistical significance is indicated as follows: ***p ≤ 0.001, **p ≤ 0.01, *p < 0.05.

### Post-infection cytokine responses reflect synergistic immune modulation between trained and acquired response

**Figure 4:**
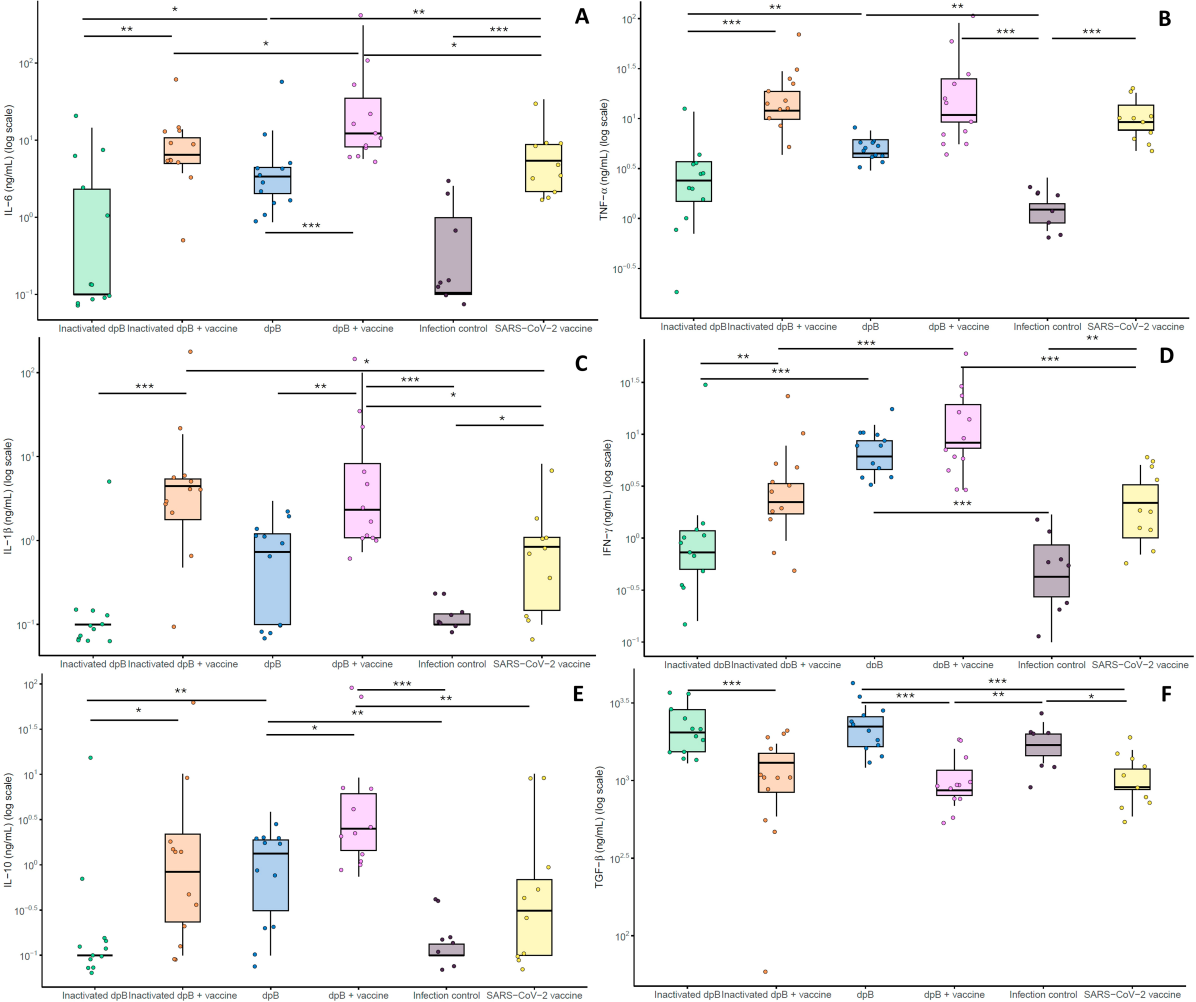
Graphical logarithmical representation (log ng/mL) for post-infection cytokines in animals sacrificed at 3–4 days post-infection (dpi) both pro-inflammatory: IL-6 **(A)**, TNF- α **(B)**, Il-1β **(C)**, INF-γ **(D)** and anti-inflammatory: IL-10 **(E)**, TGF-β **(F)** for each of the experimental groups all of them infected with SARS-CoV-2: Inactivated dpB (group 1), inactivated dpB - SARS-CoV-2 (group 2), dpB (group 3), dpB - SARS-CoV-2 vaccine (group 4), infection control group (group 5) and SARS-CoV-2 vaccine group (group 6). Statistical significance is indicated as follows: ***p ≤ 0.001, **p ≤ 0.01, *p < 0.05.

The levels for each of the cytokines at 3–4 dpi obtained were similar to the concentration obtained in the pre-infection sampling. Those receiving the live form of dpB with the SARS-CoV-2 vaccine (group 4) showed elevated IFN-γ and IL-6 levels when comparing to inactivated dpB - SARS-CoV-2 vaccine (group 2) (M-W; p_b_<0.001, p_b_=0.033) and only-SARS-CoV-2 vaccinated animals (group 6) (M-W; p_b_<0.001, p_b_=0.014) (**Figures 4A, D**). For IL-1β and IL-10, animals receiving both live dpB and SARS-CoV-2 vaccine showed significantly higher levels than SARS-CoV-2 vaccinated-only animals (M-W, p_b_ = 0.017; M-W, p_b_=0.007) (**Figures 4C, E**) reflecting an enhanced cytokine response likely associated with the synergistic effects of trained and adaptive immunity. In this case, for TNF-α cytokine, the highest levels were obtained for both groups combining immunomodulator and SARS-CoV-2 vaccine (groups 2 and 4) (**Figure 4B**).

**Figure 5:**
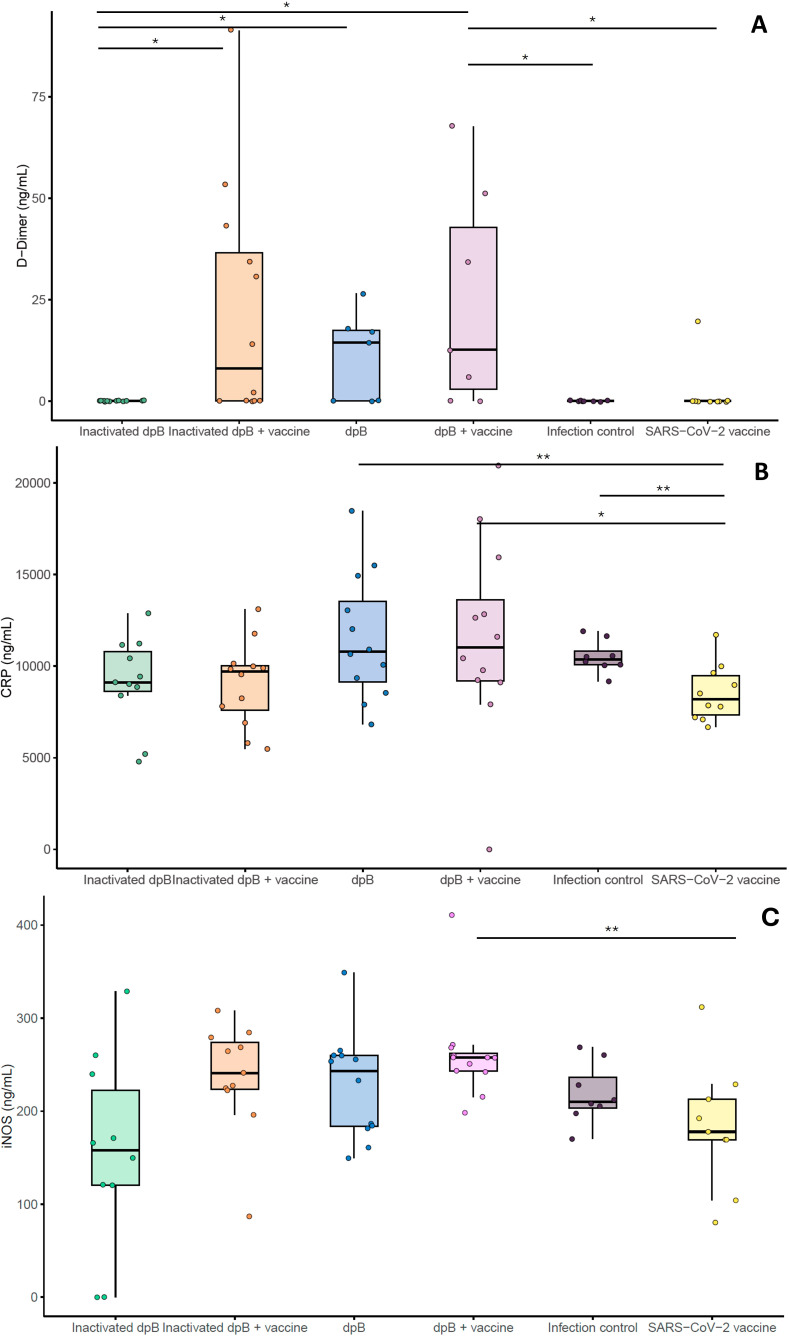
Graphical representation of inflammatory and coagulation markers (ng/ml): D-dimer **(A)**, C-reactive protein **(B)** and iNOS **(C)** from the different experimental groups: inactivated dpB (group 1), Inactivated dpB-SARS-CoV-2 vaccine (group 2), dpB (group 3), dpB-SARS-CoV-2 vaccine (group 4), Infection control (group 5) and SARS-CoV-2 vaccine (group 6) in 3–4 days post infection (dpi). Statistical significance is indicated as follows: ***p ≤ 0.001, **p ≤ 0.01, *p < 0.05.

Regarding to inflammatory markers, plasma samples collected on 3–4 dpi showed that animals from the group receiving both live dpB and SARS-CoV-2 vaccine showed higher values (mg/mL) of D-dimer, CRP and iNOS when compared to infection SARS-CoV-2 vaccinated group (M-W; p_b_=0.042, p_b_=0.021, p_b_=0.003) (**Figures 5A–C**). For animals developing the disease no significant differences were obtained comparing these experimental groups.

### Brain viral loads were lower in dpB and SARS-CoV-2 vaccinated animals developing COVID-19

**Figure 6:**
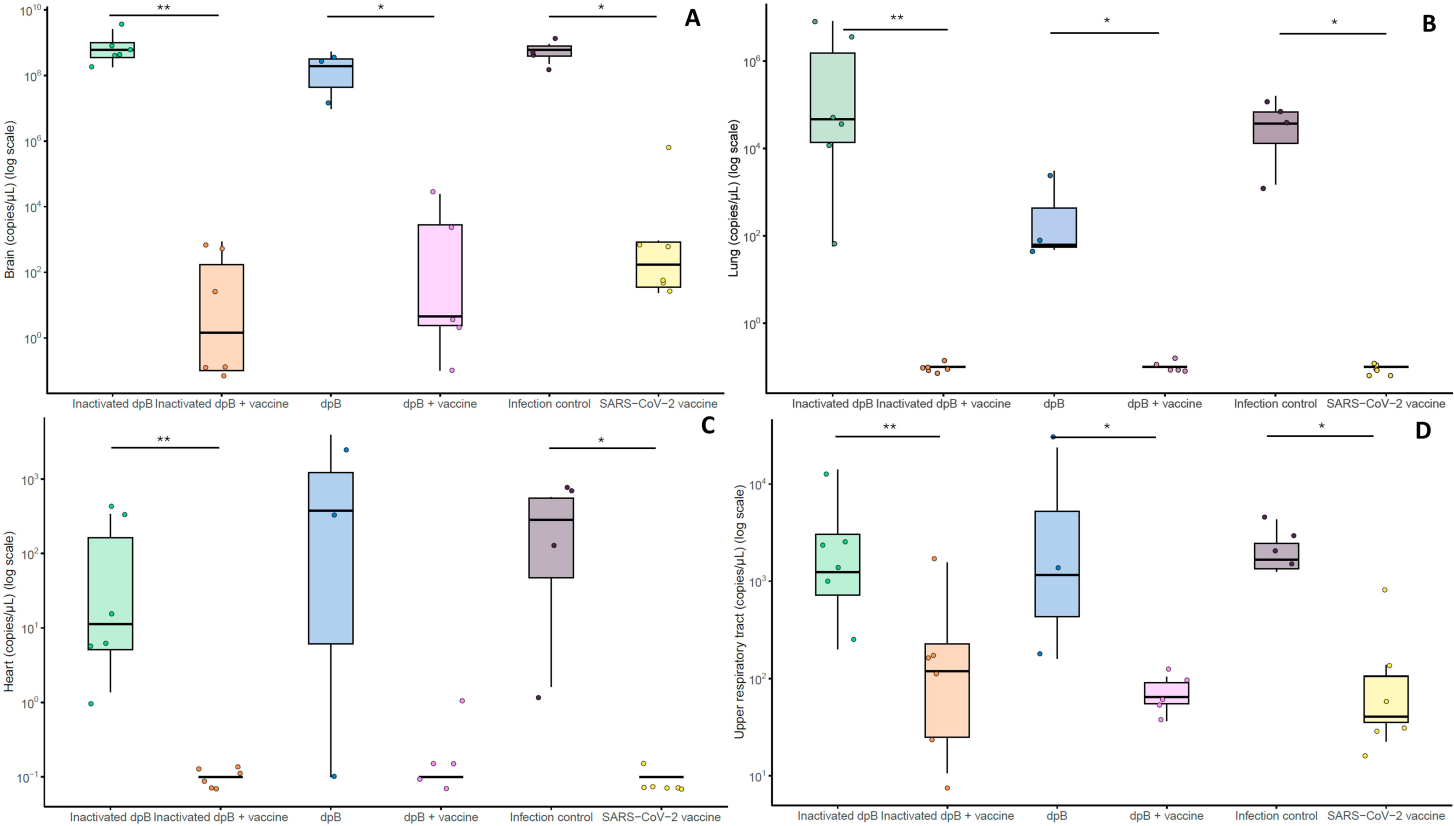
Graphical representation of the logarithm of copies per microliter of purified RNA corresponding to the viral loads in the brain **(A)**, lungs **(B)**, heart **(C)**, and upper respiratory tract **(D)** for the animals that developed the disease from group 1 to 6. Statistical significance is indicated as follows: ***p ≤ 0.001, **p ≤ 0.01, *p < 0.05.

In brain tissue, although no significant differences were obtained (K-W; p>0.05), the lowest viral loads were found in the live dpB + SARS-CoV-2 vaccine group, followed by the inactivated dpB - SARS-CoV-2 vaccine group, and finally the vaccinated animals (**Figure 6A**). In pulmonary and cardiac tissues, all vaccinated groups exhibited undetectable viral loads (**Figures 6B, C**). With respect to the upper respiratory tract including turbinates and trachea, a similar trend was observed as in brains, but with comparatively lower viral loads, i.e., vaccinated animals had the lowest viral loads although non-statistical differences were observed (K-W, p>0.05) (**Figure 6D**).

### DpB stimulation in combination with the SARS-CoV-2 vaccine reduced the severity of lesions in the brain and lungs

In line with viral loads results in tissues, further histopathological examination highlighted the higher protective effects of combining dpB with the SARS-CoV-2 vaccine. In the brain, acute lesions characteristic of SARS-CoV-2 (primarily consisted of moderate to severe non-suppurative meningoencephalitis and neuronal degeneration, characterized by cytoplasmic vacuolization with a pyknotic and eccentric nucleus) were observed. Additionally, moderate white matter tract myelin sheath vacuolation, increased glial cell proliferation, vasculitis, and perivascular hemorrhages were frequently observed.

**Figure 7:**
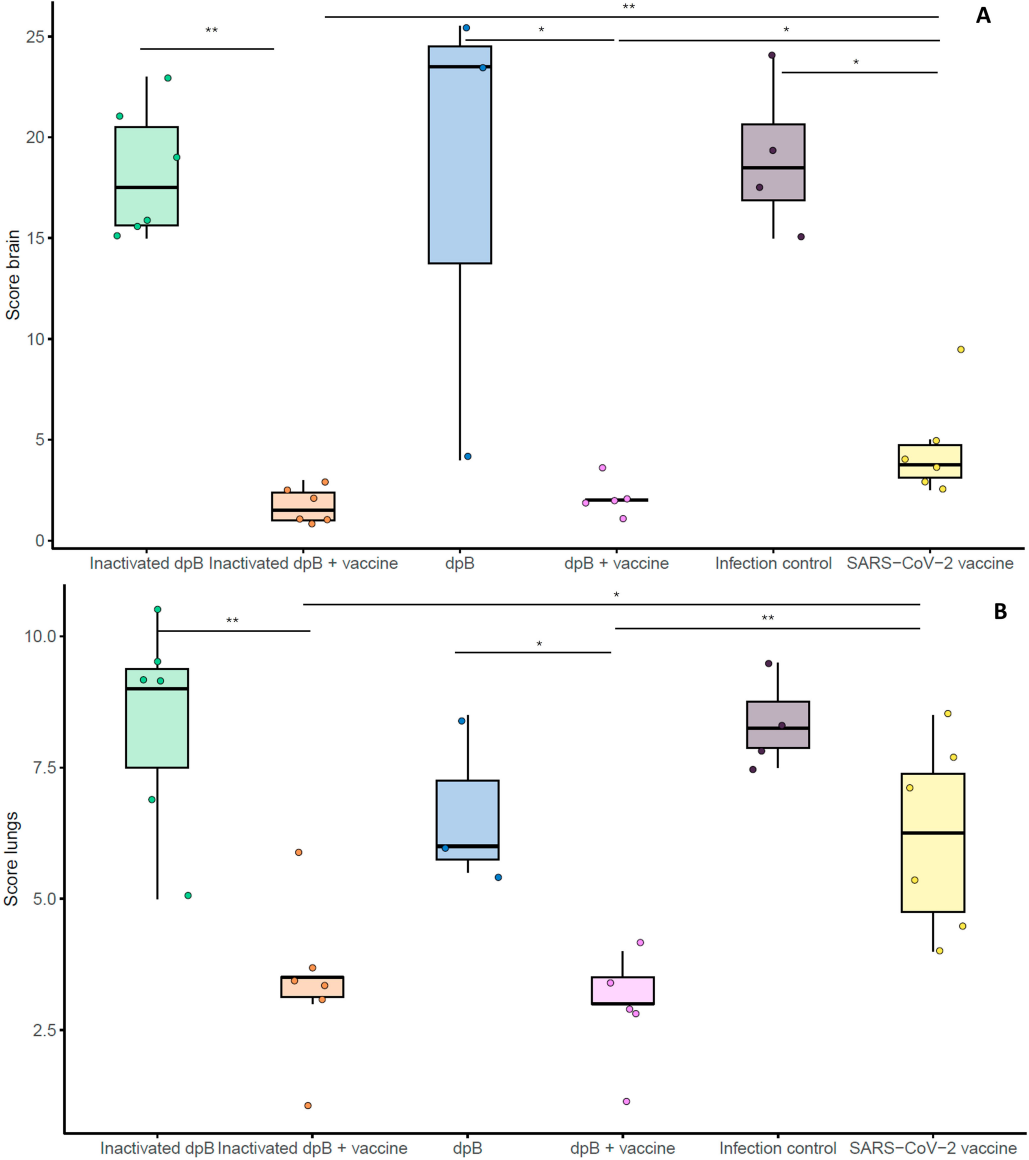
Box plot graphical representation of the median histopathological scores obtained for brain **(A)** or lung **(B)** when developing the SARS-CoV-2 disease for the different experimental groups: inactivated dpB (group 1), inactivated dpB-SARS-CoV-2 vaccine (group 2), dpB (group 3), dpB-SARS-CoV-2 vaccine (group 4), infection control (group 5) and SARS-CoV-2 vaccine (group 6). Statistical significance is indicated as follows: ***p ≤ 0.001, **p ≤ 0.01, *p<0.05.

**Figure 8:**
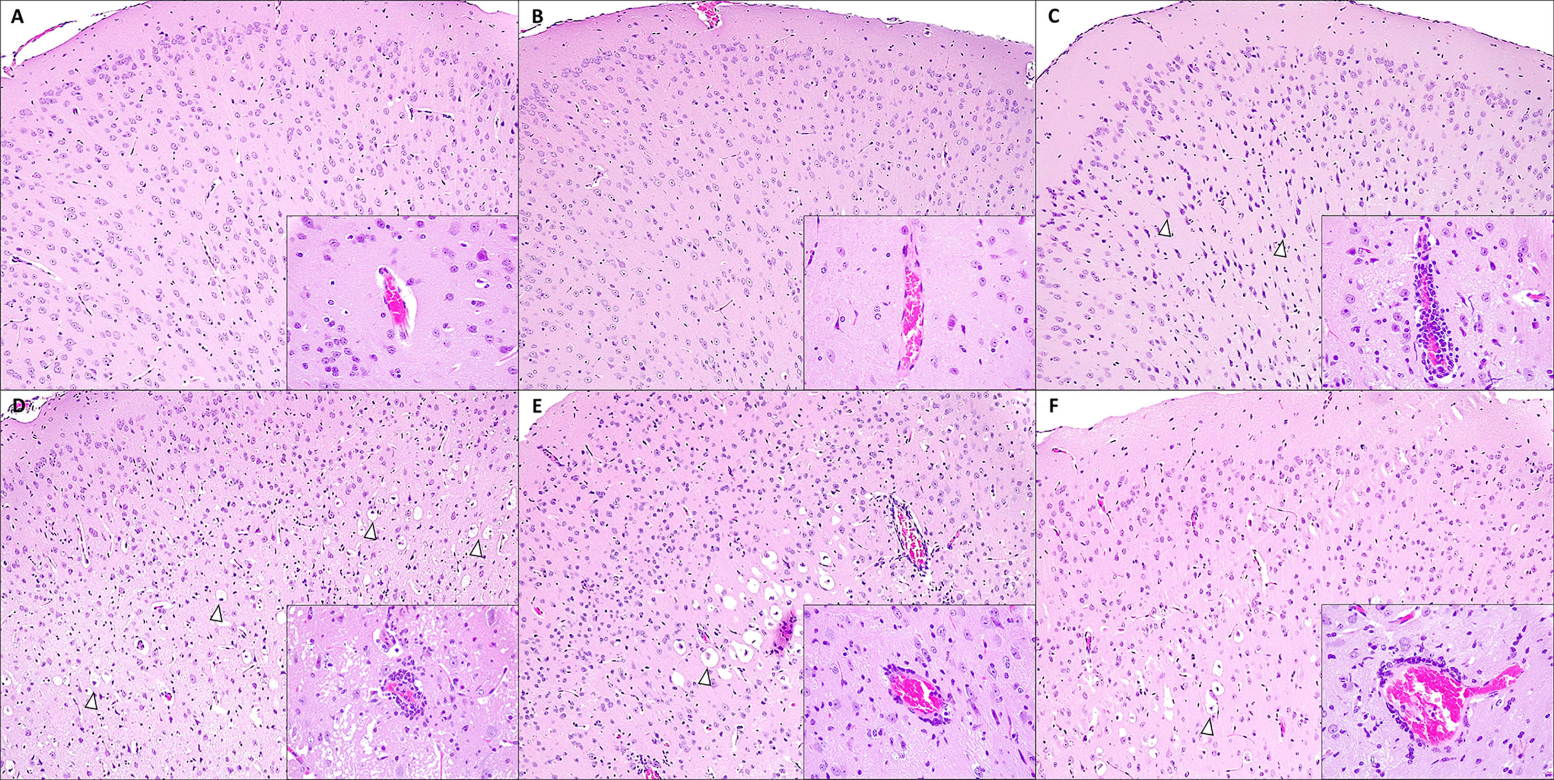
Microscopic brain lesions in the motor cortex in mice from: dpB - SARS-CoV-2-vaccine (group 4) at 9 days post infection (dpi) **(A)**; inactivated dpB - SARS-CoV-2-vaccine (group 2) at 9 dpi **(B)**; SARS-CoV-2- vaccine (group 6) at 9 dpi **(C)**; dpB (group 3) at 7 dpi **(D)**; inactivated dpB (group 1) at 8 dpi **(E)**; infection control (group 5) at 7 dpi **(F)**. A,B: absence of lesions with a normal glial cell population number; Hematoxilin-Eosin (HE), 10x. Inset: no lesion is observed in the blood vessel; HE, 40x. **(C)** Moderate number of shrunken basophilic neurons (arrowheads) and mild to moderate increase of glial cells are observed; HE, 10x. Inset: moderate to severe perivascular lymphocytic cuffs are shown; HE, 40x. **(D)** Severe number of neurons, diffusely located, with cytoplasmic balloningballooning and shrunken red nuclei (neuronal degeneration) (arrowheads) and moderate increase of glial cells are present; HE, 10x. Inset: moderate to severe perivascular lymphocytic cuffs and neuroparenchymal spongiosis. HE, 40x. **(E, F)** Moderate number of neurons, focally located, with neuronal degeneration (arrowheads) and mild to moderate increase of glial cells can be observed; HE, 10x. Inset: moderate perivascular lymphocytic cuffs. HE, 40x.

DpB-SARS-CoV-2 vaccine (group 4) [median histopathological score (MHS)=2.0] and inactivated dpB-SARS-CoV-2 vaccine group (group 2) (MHS=1.5) showed an absence of brain lesions characteristic of SARS-CoV-2 infection at 7–9 dpi (**Figure 7A**). In comparison, SARS-CoV-2 vaccinated group (group 6) also did not present typical lesions (MHS=3.75), except for one case (HS=9.5) (**Figure 7A**), which had moderate perivascular lymphocytic cuffs and a moderate increase of shrunken neurons and glial cells, at 9 dpi. The total score in group 6 was significantly higher than groups 4 and 2 (M-W, p= 0.017, p=0.009) (**Figures 8A–C**).

In the lungs, acute lesions characteristic of SARS-CoV-2 included bronchointerstitial pneumonia, characterized by infiltration of mononuclear cells (mainly macrophages plasma cells and lymphocytes) and occasional neutrophils around bronchioles and blood vessels, mild to moderate patchy septal thickening; type II pneumocyte hyperplasia, perivascular lymphocytic cuffs, microhemorrhages and alveolar edema.

**Figure 9:**
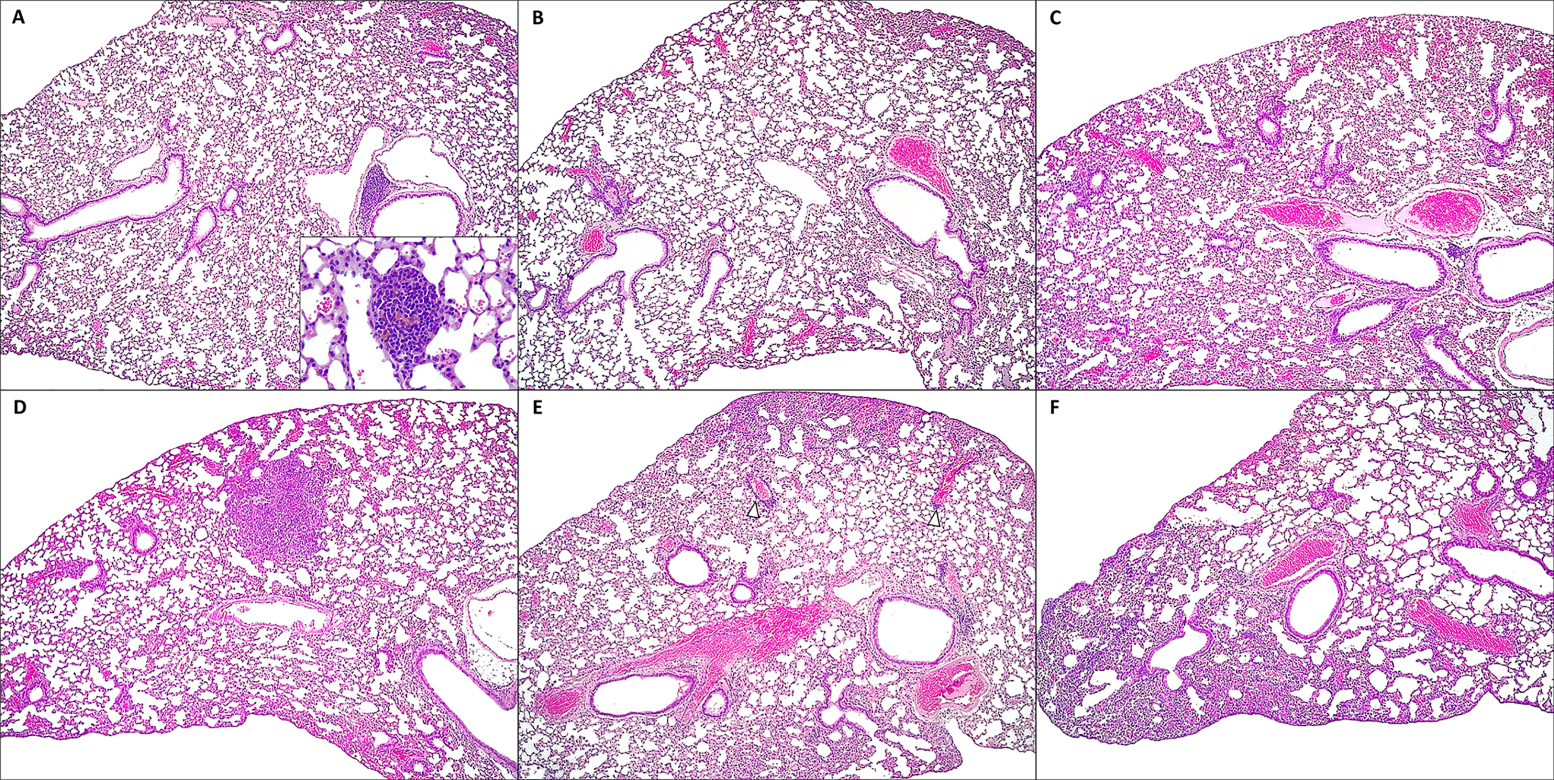
Microscopic lung lesions in mice from: dpB - SARS-CoV-2-vaccine (group 4) at 9 days post-infection (dpi) **(A)**; inactivated dpB - SARS-CoV-2-vaccine (group 2) at 9 dpi **(B)**; SARS-CoV-2- vaccine (group 6) at 9 dpi **(C)**; dpB (group 3) at 7 dpi **(D)**; inactivated dpB (group 1) at 7 dpi **(E)**; control infection (group 5) at 7 dpi **(F)**. **(A)** Minimal thickening of alveolar septum with moderate hyperplasia of the bronchus-associated lymphoid tissue; Hematoxilin-Eosin (HE), 10x. Inset: round lymphoid formation composed of mononuclear cells and small capillaries, located at the alveolar septum; HE, 40x. **(B)** Minimal thickening of alveolar septum; HE, 10x. **(C)** Minimal to mild thickening of alveolar septum; HE, 10x. **(D-F)** Mild to moderate interstitial pneumonia with patchy thickening of alveolar septum, with occasional perivascular lymphocytic inflammation (arrowheads); HE, 10x.

Similarly, reduced severity of lung lesions at 9 dpi was observed in groups that combine the vaccine with dpB. Live dpB +SARS-CoV-2 vaccine (group 4) (MHS=3.0) and inactivated dpB-SARS-CoV-2 vaccine (group 2) (MHS=3.5) animals characterized by minimal interstitial pneumonia and thickening of the alveolar septa; whereas SARS-CoV-2 vaccine group (group 6) (MHS=6.25) showed the same lesions, but slightly more pronounced mainly when compared to groups 4 and 2 (M-W, p_b_=0.004, p_b_=0.015) (**Figures 7B**, **9A–C**). Group 4 was notable for presenting hyperplasia of the bronchus and bronchioles-associated lymphoid tissue (BALT). Additionally, it exhibited round lymphoid formations, characterized by the aggregation of lymphoplasmacytic cells and macrophages, associated with small blood vessels and located in the alveolar interstitium, not adjacent to the bronchi or bronchioles [compatible with inducible BALT (iBALT) or tertiary lymphoid organs (TLO)] (**Figure 9A**).

### The immunomodulator format (live *vs.* inactivated) matters on its ability to induce trained immunity

#### DpB induced higher cytokine levels response before and after SARS-CoV-2 infection compared to inactivated dpB

Prior to infection, animals that had been stimulated with live dpB (group 3) had higher IFN-γ (**Figure 3D**), IL1-β (**Figure 3C**) TNF-α (**Figure 3B**) and IL-6 (**Figure 3A**) values than the group of inactivated dpB stimulated animals (group 1) or the infection controls (that had not been stimulated) (group 5). Moreover, the values for IFN-γ cytokine for the live dpB stimulated group (median ± standard deviation) (7.86 ± 17.27 ng/ml) were almost as high as in the group combining live dpB stimulation with the SARS-CoV-2 vaccine (8.70 ± 79.75 ng/ml) (**Figure 3D**). For the anti-inflammatory cytokine TGF-β, these groups of animals without vaccine had higher values than their respective groups with SARS-CoV-2 vaccine present (**Figure 3F**). This is traduced in higher levels in the inactivated dpB group versus the inactivated dpB – SARS-CoV-2 group, live dpB higher than live dpB with SARS-CoV-2 vaccine and the infection control group significantly higher values than the SARS-CoV-2 vaccine only group. Similarly, IFN-γ and IL-10 values were higher for animals inoculated with live dpB when compared to both inactivated dpB and infected controls at 3–4 dpi (**Figures 4D, E**). Higher values of IL-6 were obtained in live dpB inoculated group when compared to inactivated dpB animals (**Figure 4A**). Otherwise, TFN-α levels were significantly higher in live dpB-inoculated animals in comparison to both the inactivated dpB inoculated animals and the infected control group (**Figure 4B**).

#### DpB does not induce protection itself against SARS-CoV-2 infection although live format has different impact depending on the tissue

For mortality rates, in group 1 (inactivated dpB) rose to 100% (6/6); 66.6% (2/3) in group 3 (dpB) and 100% (4/4) in infection control group 5 (infection control group). Animals that became sick started to show mild clinical signs such as reduced activity or moderate weight loss at 5 dpi. On days 6, 7 and 8 dpi these clinical signs were joined by increased depression or respiratory rate, and in some cases reaching dyspnea, absence of activity, semi- closed eyes, tousled hair, and severe weight loss (**Figure 2**).

In the unvaccinated groups, the highest viral load was observed in the lungs of inactivated dpB stimulated animals (group 1) (5x10^4^ ± 3.39x10^6^ copies/µl) and infection control group (group 5) (3.90x10^4^ ± 6.77x10^4^ copies/µl) and lastly animals stimulated solely with dpB (group 3) (**Figure 6B**). Regarding heart viral loads, higher values were obtained for group 3 (3.78x10^2^ ± 2.15x10^3^ copies/µl), followed by group 5 (3.46x10^2^ ± 2.88x10^2^ copies/µl), being group 1 the one with the lowest viral loads in the heart ± (**Figure 6C**). However, significant differences for all tissues were only present when comparing each group with its parallel group adding SARS-CoV-2 vaccine. In this way, significant differences were observed for brain (**Figure 6A**), lung (**Figure 6B**), heart (**Figure 6C**) and upper respiratory tract (**Figure 6D**) with higher levels of copies/μl in inactivated dpB (group 1) versus inactivated dpb-SARS-CoV-2 vaccine (group 2), dpB group (group 3) versus dpB-SARS-CoV-2 vaccine (group 4) (except for heat values) and infection control group (group 5) against SARS-CoV-2 vaccine group (group 6) (M-W, p<0.05).

Animals from the infection control group (group 5) exhibited severe acute brain lesions characteristic of SARS-CoV-2 infection. In group 3 (live dpB), two animals (2/3 cases) presented the most severe brain lesions at 7 dpi (MHS=23.5), observing a more extensive neuronal degeneration. All animals of groups 1 (inactivated dpB) (MHS=17.5) and 5 (infection control) (MHS=18.5) had similar acute brain lesions at 6–8 dpi, being more intense at 8 dpi. (**Figures 8D–F**).

In non-vaccinated animals (groups 1, 3 and 5), lung lesions characteristic of SARS-CoV-2 infection were classified as mild to moderate acute lung lesions. Although the differences between groups were minimal, a lower severity was observed in live dpB group (MHS=6.0) compared to inactivated dpB group (MHS=9.0) and infection control group (MHS=8.25) (**Figure 9**), frequently presenting multifocal areas of broncointerstitial pneumonia and perivascular lymphocytic cuffs.

## Discussion

Abundant research assessed the efficacy of BCG against different diseases (12, 13, 35, 36). However, in the case of SARS-CoV-2, there are controversial results (17, 18, 20, 21, 25). This study investigated the potential impact of a live and inactivated dpB (pig-derived BCG) stimulation on the COVID-19 protection induced by SARS-CoV-2 vaccination. We found that combining trained and adaptive immunity can enhance protection against SARS-CoV-2 and that the format of the trained immunity inducer (live/inactivated) may alter its immunomodulatory effect in the murine model.

Consistent with previous research (20, 21, 25), our findings underscore the limitations of mycobacterial trained immunity inducers on their own to modulate efficient protection against SARS-CoV-2. In our study, the format of the immunomodulator was proven to be of relevance in this regard. A key factor appears to be the heightened by pro-inflammatory cytokine response in animals inoculated with live dpB. The increased levels of IL-1β, TNF-α and IL-6 in these animals suggest that trained immunity mechanisms, characterized by epigenetic and metabolic reprogramming of monocytes and macrophages (1) or NK cells (3), may enhance early innate immune responses upon viral exposure. This is consistent with prior studies showing that BCG-trained monocytes and alveolar macrophages exhibit increased responsiveness to secondary infections (37), leading to a more effective early immune response against respiratory pathogens.

Furthermore, the lower viral loads in the lungs of dpB-stimulated animals supports prior evidence suggesting that BCG stimulation can confer non-specific protection against respiratory infections (9) by enhancing alveolar macrophage function and promoting rapid clearance of pathogens (38). Notably, BCG has shown to induce local lung immunity via the recruitment of monocytes and macrophages, which may explain the observed reductions in pulmonary viral loads (39). This effect could also be attributed to the induction of autonomous alveolar macrophages (AMs) with a trained immunity phenotype (40, 41) characterized by high glycolytic activity and enhanced cytokine release. Interestingly, recent findings suggest that the establishment of memory AMs in the respiratory mucosa requires the contribution of effector CD8+ T cells throughout IFN-γ, consistent with higher levels in dpB group in this study, during the early stages of the antiviral response (41). Histopathological findings further reinforce this trend, presenting animals that received live dpB slightly less severe lung lesions compared to those treated with inactivated dpB or non-stimulated controls. However, in the case of lesions observed at the brain level, although slightly, the animals stimulated with live dpB showed more severe lesions than the inactivated dpB or control group. This more severe brain lesions, agrees with what we observed in a previous study inoculating BCG prior to SARS-CoV-2 infection (25). Although some studies demonstrate some alleviation of neuroinflammation due to BCG (42), these results suggest that live dpB is relatively protective at the pulmonary level but not at the brain level when it comes to subsequent infection with SARS-CoV-2.

Previous studies have already demonstrated that inducers of trained immunity, in combination with specific vaccines, enhance protection against the pathogen in concern (7, 43). For this reason, we carried out the evaluation of impact of the combination of dpB in different formats together with a subsequent specific vaccination against SARS-CoV-2 against the exposition to the virus. While SARS-CoV-2 vaccination on its own provides strong protection demonstrated by practically no clinical signs and no mortality, our findings indicate that its combination with dpB-trained immunity inducer significantly amplifies specific immune response which is reflected on pro-inflammatory response, viral loads and histological lesions. This was particularly evident in the heightened levels of pro-inflammatory cytokines observed in animals that received live dpB together with the SARS-CoV-2 vaccine (See **Figure 1**). Specifically, IFN-γ, IL-6 and IL-1β were significantly elevated in these animals compared to those receiving only the SARS-CoV-2 vaccine. These results align with previous studies showing that trained immunity induced by BCG, primes innate immune cells to respond more robustly to secondary challenges, leading to enhanced cytokine production upon infection (1). The elevation of IL-6 and IL-1β further supports this, as these cytokines are essential for initiating effective inflammatory responses and recruiting immune cells to sites of infection (20). Importantly, the increased of anti-inflammatory IL-10 levels observed in these animals suggest that, despite the strong pro-inflammatory response, mechanisms of immune regulation were also active, potentially preventing an exacerbated inflammatory response (44).

The presence of significantly higher IFN-γ levels in animals receiving live dpB alongside the COVID-19 vaccine is particularly relevant, given its role in antiviral defense and immune regulation (45). While this cytokine is not directly associated with trained immunity, it plays a crucial role in both innate and adaptive immune responses against bacteria, cancer and viruses (46), primarily by promoting macrophage activation, enhancing antigen presentation via MHC upregulation (47), and stimulating the cytotoxic activity of NK and T cells (37, 48). Some studies propose that macrophages can act as direct sources of IFN-γ, producing both local and systemic signals that enhance antimicrobial defenses (45). This macrophage-derived IFN-γ has been linked to bacterial growth control (49), suggesting a broader immunoregulatory function that extends beyond its traditional role. In our study, the significant increase in IFN-γ levels in animals combining trained and adaptive immunity inducers suggests a potential synergistic effect, where innate immune priming via dpB enhances IFN-γ production, contributing to improved viral clearance and tissue protection. The simultaneous activation of trained and adaptive immunity may generate a more effective early immune response, contributing to better viral control and improved tissue protection. These findings further support the idea that dpB-induced trained immunity does not act in isolation but rather complements and amplifies adaptive immune mechanisms, ultimately enhancing host defense against SARS-CoV-2.

In addition to the elevated cytokine levels observed in animals receiving dpB alongside the SARS-CoV-2 vaccine, significant differences were also detected in key inflammatory and coagulation-related biomarkers, including D-dimer, C-reactive protein, and inducible nitric oxide synthase. D-dimer, a fibrin degradation product, is commonly used as a marker of coagulation activation and thrombotic risk, particularly infections that trigger systemic inflammation (50). The observed increase in D-dimer levels in animals receiving dpB suggests a heightened activation of the coagulation cascade, potentially as a result of a more robust inflammatory response. This aligns with the increased levels of IL-6 and IL-1β in these animals, as both cytokines are known to upregulate tissue factor expression and promote a pro-coagulant state (50). While excessive coagulation can be detrimental, controlled activation of this pathway may contribute to immune defense by limiting pathogen dissemination and facilitating immune cell recruitment (51).

Similarly, the significantly higher CRP levels in the dpB-treated group further support the presence of an enhanced inflammatory response. CRP is an acute-phase protein produced by the liver in response to elevated cytokine levels, particularly IL-6, and serves as a key biomarker of systemic inflammation (52). Its increased expression in these animals likely reflects the amplified inflammatory signaling driven by trained immunity.

Additionally, the increased expression of iNOS in dpB-treated animals provides additional evidence of enhanced immune activation, particularly in the context of macrophage-driven responses. iNOS is expressed under inflammatory conditions in various cell types, including macrophages, where it plays a crucial role in antimicrobial defense by generating nitric oxide (NO), a potent effector molecule involved in pathogen clearance (53). The elevated iNOS levels observed in our study are consistent with the increased IFN-γ production, as this cytokine is a key inducer of iNOS expression and macrophage activation (54). This suggests that dpB not only enhances cytokine production but also promotes a more robust antimicrobial environment, potentially contributing to improved viral clearance.

Consistent with the cytokine profile, viral loads in brain tissue were lowest in animals that received both live dpB and the SARS-CoV-2 vaccine, followed by those receiving inactivated dpB with the vaccine, and finally the SARS-CoV-2 vaccinated-only group. Although differences were not statistically significant, this trend suggests that the synergy between trained and adaptive immunity may contribute to enhanced viral clearance and subsequently higher protection in tissues.

Histopathological analyses further demonstrated the higher protective effects of combining dpB with the SARS-CoV-2 vaccine compared with vaccine alone. In the brain, the severity and frequency of lesions, including non-suppurative meningoencephalitis and neuronal degeneration, were absent animals receiving the combined stimulation strategy (inactivated/live dpB + SARS-CoV-2 vaccine), on the contrary to live dpB on its own against SARS-CoV-2, which also demonstrated under other circumstances to have harmful effects (25). Similarly, lung lesions were less severe in animals that received both forms of dpB with the SARS-CoV-2 vaccine in comparison to only vaccinated animals. The reduced severity of bronchointerstitial pneumonia and alveolar edema in these groups suggest that trained immunity contributes with vaccination to limiting SARS-CoV-2-induced lung damage. One of the most notable findings in animals receiving live dpB combined with the SARS-CoV-2 vaccine was the BALT hyperplasia, but also the formation of ectopic lymphoid tissue, compatible with inducible BALT (iBALT). BALT is a secondary lymphoid tissue involved in the development of bronchopulmonary immune responses (55). On the other hand, iBALT formation is caused by response to infections or chronic inflammation (56). While its exact role in pathogenesis remains unclear, it may serve as a protective immune mechanism against viral pathogens (57). A previous study suggests that iBALT helps regulate immune responses by delaying Th2 cell accumulation and altering T cell distribution in the lungs (58); this process may attenuate pulmonary inflammation and prevent excessive tissue damage (57). Therapies promoting iBALT formation could represent a novel immunoprophylactic strategy for broad-spectrum viral protection (59). BALT development has been reported in previous studies following BCG administration supporting the hypothesis that these structures contribute to long-term immune surveillance and protection (60, 61), reinforcing the idea that BCG can stimulate the formation of organized lymphoid structures in the lungs.

In conclusion, these findings highlight the advantages of combining trained and adaptive immunity in enhancing vaccine-induced protection against SARS-CoV-2. While the SARS-CoV-2 vaccine alone provides effective protection in terms of morbidity and mortality in our murine model, its combination with dpB further amplifies immune responses which is reflected in cytokine response, viral load and histopathological lesions (particularly in brain). Further studies should aim to optimize the use of BCG in combination with specific vaccines by evaluating different formulations, dosing regimens and routes of administration. Additionally, future investigation into the mechanisms driving BALT hyperplasia and its functional role in antiviral immunity could provide valuable insights for designing novel vaccination strategies. Understanding how trained immunity may influence long-term protection could pave the way for innovative approaches that integrate broad-spectrum immune priming with targeted adaptive immunity.

## Data Availability

The original contributions presented in the study are included in the article/**Supplementary Material**. Further inquiries can be directed to the corresponding authors.
